# The phylogeographic history of *Megistostegium* (Malvaceae) in the dry, spiny thickets of southwestern Madagascar using RAD‐seq data and ecological niche modeling

**DOI:** 10.1002/ece3.8632

**Published:** 2022-02-16

**Authors:** Margaret M. Hanes, Susan Shell, Tahsina Shimu, Clarissa Crist, Salima Machkour‐M’Rabet

**Affiliations:** ^1^ 8759 Department of Biology Eastern Michigan University Ypsilanti Michigan USA; ^2^ Departamento de Conservación de la Biodiversiadad El Colegio de la Frontera Sur Chetumal Mexico

**Keywords:** dry spiny thicket, Madagascar, Malvaceae, *Megistostegium*, niche evolution, phylogeography

## Abstract

The spiny thicket of southwestern Madagascar represents an extreme and ancient landscape with extraordinary levels of biodiversity and endemism. Few hypotheses exist for explaining speciation in the region and few plant studies have explored hypotheses for species diversification. Here, we investigate three species in the endemic genus *Megistostegium* (Malvaceae) to evaluate phylogeographic structure and explore the roles of climate, soil, and paleoclimate oscillations on population divergence and speciation throughout the region. We combine phylogenetic and phylogeographic inference of RADseq data with ecological niche modeling across space and time. Population structure is concurrent with major rivers in the region and we identify a new, potentially important biogeographic break coincident with several landscape features. Our data further suggests that niches occupied by species and populations differ substantially across their distribution. Paleodistribution modeling provide evidence that past climatic change could be responsible for the current distribution, population structure, and maintenance of species in *Megistostegium*.

## Graphical abstract

We investigate drivers of plant diversification in the spiny thickets of southwestern Madagascar. Phylogeographic inference of RADseq data and ecological niche modeling across space and time in the regional endemic genus *Megistostegium* (Malvaceae) identifies population structure concurrent with major rivers and evidence that past climatic change could be responsible for the current distribution and maintenance of species in the genus.

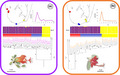

## 1 INTRODUCTION

One important aim of evolutionary biology is to understand the processes responsible for speciation, and one of the most exciting places to study species is on Madagascar. The island is renowned for its diverse and highly endemic biota and has been identified as a model region to understand species diversification (Vences et al., 2009). The extraordinary biodiversity from the island’s humid forests have been explained by several forces, including elevation gradients and river barriers (Boumans et al., 2007; Goodman & Ganzhorn, 2004; Louis et al., 2005; Pastorini et al., 2003; Vieites et al., 2006; Wollenberg et al., 2008). River basins have further been suggested to act as refugia (Mercier & Wilmé, 2013; Wilmé et al., 2006), and isolating barriers, when past aridification events forced forests to contract around rivers. In contrast with many of these studies, southwestern Madagascar has ephemeral rivers, little to no elevational change, and few studies have explored the processes driving diversification (but see Florio & Raxworthy, 2016; Pabijan et al., 2015; Shi et al., 2013). Though morphological adaptations in response to climate have been explored in southwestern Madagascar (Evans et al., 2014), few other hypotheses exist for diversification in dry adapted plant taxa in this region (Ganzhorn et al., 2001; Nicoll & Langrand, 1989).

The dry spiny thickets of southwestern Madagascar (as defined by Aronson et al., 2018) occupy a sandy strip along the southwestern coast of the island and cover an area of approximately 16,000 km^2^. This semiarid zone is located in one of the oldest biomes on the island (Buerki et al., 2013) and boasts some of the highest plant endemism on Madagascar (Davis et al., 1994; Jolly et al., 1984; Phillipson, 1996): 48% of plant genera and 95% of plant species occurring in the ecoregion are endemic to the island. The tree flora recorded from this zone is similarly exceptional with 89% of the tree species endemic to Madagascar and more than 20% of tree species endemic to the dry spiny thickets (Aronson et al., 2018). Despite these remarkable figures and the fact that dry, deciduous forests are among the most threatened biomes of the world (Janzen, 1988), the spiny thickets remain understudied (Moat & Smith, 2007; Waeber et al., 2015).

Understanding how past climate has altered biotic distributions may be particularly important in understanding divergence patterns throughout southwestern Madagascar. Aridity increased substantially throughout the Late Pleistocene and Holocene and subsequently expanded the xeric thickets of southwestern Madagascar (Clarke et al., 2006). Such extreme changes in climate are likely to strongly impact coastal species. These recent changes are especially compatible in the exploration of population divergence within species as frequent range shifts might allow for the reinforcement of reproductive barriers and/or provide opportunities for secondary contact and potential hybridization (Dobzhansky, 1940; Kay & Schemske, 2008; Liu et al., 2014; Rieseberg et al., 2003). The dry spiny thicket has been identified as a center of endemism and is bisected by a watershed proposed to act as a refugium for mesic species (Wilmé et al., 2006). Such low elevation watersheds are expected to have more extreme ecological shifts and a greater impact on habitat isolation than watersheds at higher elevations (Wilmé et al., 2006). Contrary to traditional refugia models, one might expect watersheds to act as an important barrier to dry‐adapted taxa. Increasing aridity would remove the barrier and allow for population expansion or increased population connectivity.

**FIGURE 1:**
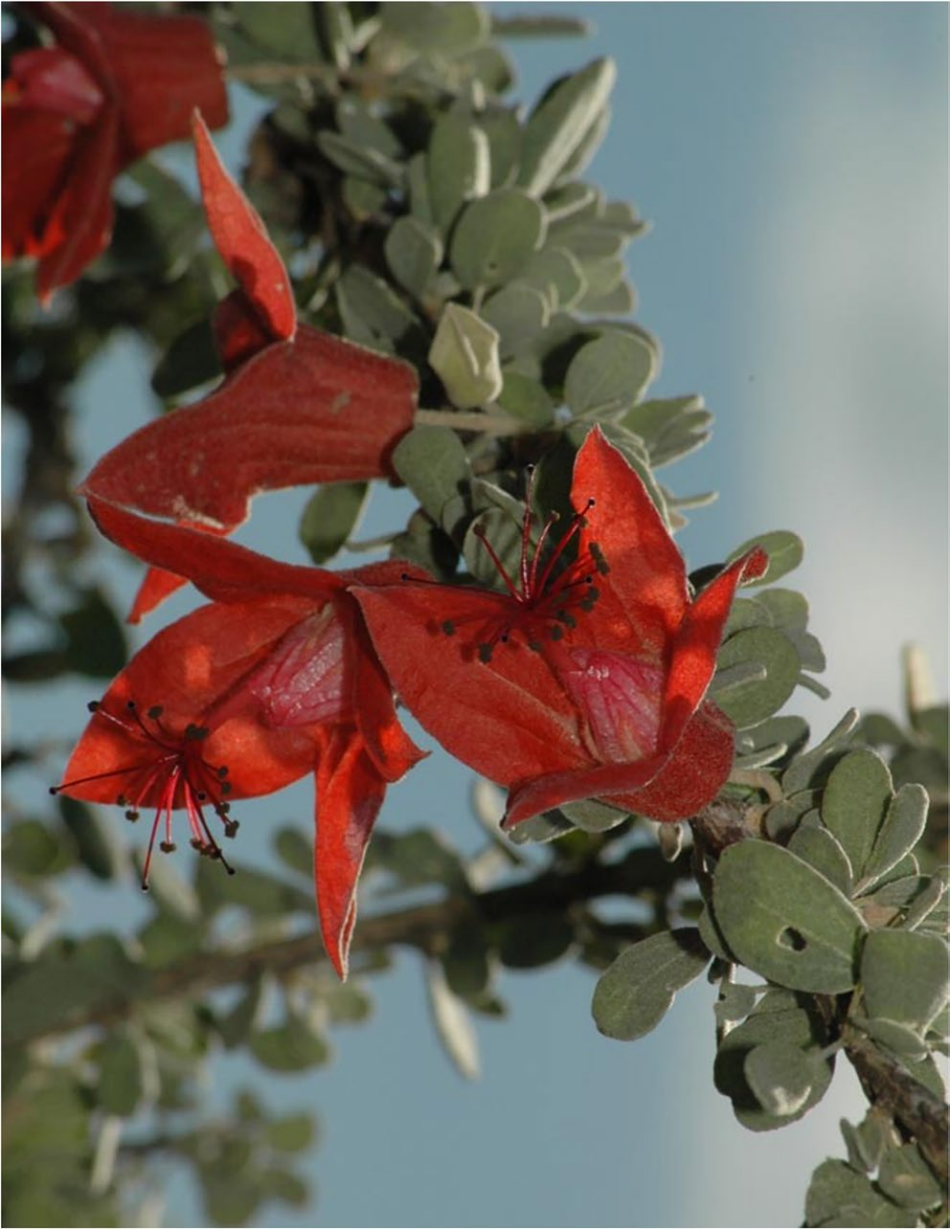
*Megistostegium microphyllum* (Malvaceae). Photo credits: Margaret Hanes

The Malvaceae family is the second most species‐rich family in the spiny thickets of southwestern Madagascar (Aronson et al., 2018). *Megistostegium* Hochr. (Figure 1) is one of nine Malvaceous genera present in the region and the only one wholly endemic to the spiny thickets. As such, the genus represents an extraordinary group to explore diversification in Malagasy plants in this biodiversity hotspot. The three species of *Megistostegium* (M. *microphyllum* Hochr., *M*. *nodulosum* (Drake) Hochr., and *M*. *perrieri* Hochr.) are estimated to have diverged sometime in the Pliocene (~5MYA; Koopman & Baum, 2008). Each species is morphologically distinct in habit (tall shrub, tree, and prostrate shrub), floral, and leaf morphologies (Hochreutiner, 1955; Koopman, 2005, 2011). Species of *Megistostegium* grow together in populations throughout southern Madagascar and evidence for interspecific hybridization is present at these sites in the form of limited prezygotic isolating barriers as assessed by hand pollination (Koopman, 2008) and the presence of infrequent morphological intermediates (Koopman, 2011). All three species grow within 100–200 m of one another at the Special Reserve of Cap Sainte Marie (Koopman, 2008, 2011) where they have overlapping flowering periods, only one potential pollinator (the Souimanga sunbird, *Cinnyris sovimanga* Gmelin), and putative hybrids have been found between two species pairs (*M*. *microphyllum* and *M*. *nodulosum*, *M*. *microphyllum* and *M*. *perrieri*). Despite these factors and demonstrated gene flow at the molecular level at Cap Sainte Marie (Koopman & Baum, 2010) the three species of *Megistostegium* maintain their morphological identity for the most part in sympatry as measured by morphology at two spatial levels (across its range in southwestern Madagascar and in sympatry at Cap Sainte Marie; Koopman, 2008, 2011; Koopman & Baum, 2010).

Given the recent origin of the genus and noted lack of complete reproductive isolation inference of relationship between the species in *Megistostegium* has proven difficult (Koopman & Baum, 2010). Population‐level data previously collected in *Megistostegium* from across its distribution indicate that the species show little genetic variation at the loci investigated: gene genealogies constructed from four nuclear and one chloroplast genes found variable levels of species monophyly and no exclusive species monophyly (Koopman & Baum, 2010). structure analyses and a series of AMOVA for six microsatellites suggested that genetic variation comes mostly from within populations and species rather than between them (Callewaert, 2014). More data are needed to understand relationships among these young species as well as population structure within species. RAD sequencing has proven to be an effective and robust method to resolve species level questions, even those with interspecific gene flow, and phylogeographic patterns (Eaton & Ree, 2013; Hipp et al., 2014; Park & Donoghue, 2019).

Here, we use RADseq to investigate relationships between species and populations of *Megistostegium* in an effort to begin to understand diversification patterns across southwestern Madagascar. We then explore patterns of niche evolution in species, and in populations, to identify geographic barriers and to understand how climate and soil might contribute to patterns of population divergence in *Megistostegium*. Specifically, we build population trees, explore the similarity of climatic niches between species and regions, and assess how landscape features and climatic change may have driven the current niche diversity and distribution in *Megistostegium*.

## 2 MATERIALS AND METHODS

### 2.1 Sampling and Genomic library preparation

**FIGURE 2:**
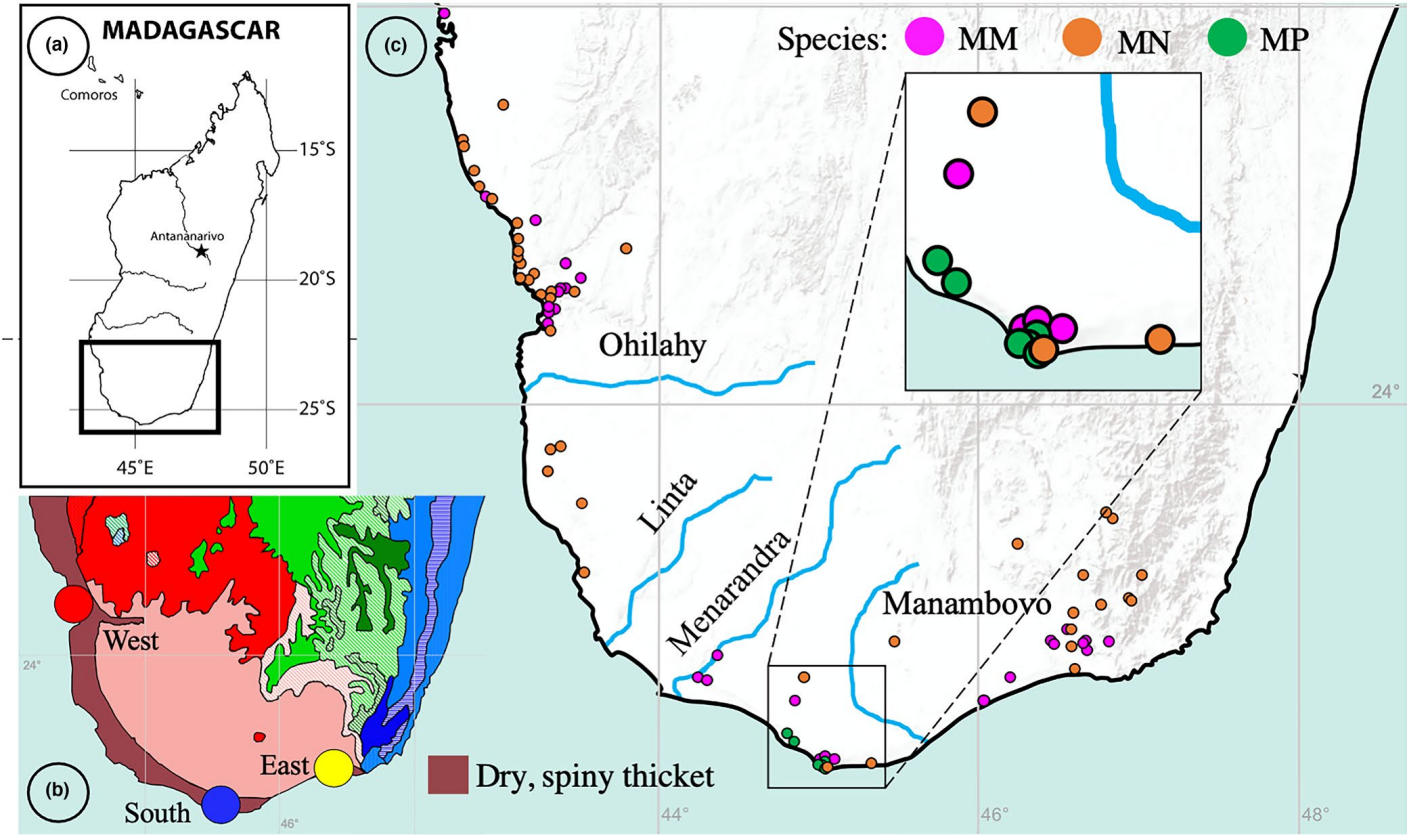
(a) Study region in southwestern Madagascar, (b) Distribution of subarid, dry, spiny thicket (bioclimate map after Cornet, 1974, obtained from Missouri Botanical Garden Madagascar gazetteer (http://www.mobot.org/mobot/gazetteer/)) and location of the three major collection regions in this study (circles in primary colors), (c) Point locations representing species distributions used for niche modeling and rivers that bisect the southern slopes of southwestern Madagascar (after Aldegheri, 1972)

A total of 89 *Megistostegium* individuals from the three species (represented in secondary colors on figures) were genotyped across southern Madagascar (Figure 2) from three geographic regions (East, West, South, Figure 2b; represented in primary colors on figures) and 12 populations (Table 1). Between 12 and 40 individuals are represented from each species with between 4 and 11 individuals from each population (Table 1). Genomic DNA from silica‐dried field‐collected leaf material was extracted using a modified CTAB method (Alverson et al., 1999), further cleaned with a DNeasy kit (Quiagen) and quantified using a Qubit 2.0 Fluorometer (Thermofisher).

**TABLE 1:** Locality data and one representative voucher specimen for species and populations sampled

Species	Region	Population name	N	Latitude	Longitude	Locality	Voucher Information (Herbarium deposited)
*Megistostegium (abbrev.)*							
*M*. *perrieri* (MP)	South1	MP1	6	−25°35′41.5″S	45°07′29.1″E	Madagascar, Cap Ste. Marie Special Reserve. At cap, south of lighthouse	*M*. *Hanes* 483 (EMC, TAN)
	South2	MP2	6	−25°25′47.5″S	44°58′26.8″E	Madagascar, Cliffs above Lavanono	*M*. *Hanes* 362 (MO, P, TAN, WIS)
Total		2	12				
	South	MMcsm2	11	−25.35′05.5″S	45.09′26.3″E	Madagascar, Cap Ste. Marie Special Reserve. 8 km from lighthouse. En route from ANGAP to cap	*M*. *Koopman* 360 (MO, P, TAN, WIS)
	East	MMAMW2	7	−25°00′29.5″S	46°27′29.0″E	Madagascar, Fort Dauphin area, 8 km west of Ambosary	*M*. *Hanes* 493 (EMC, MICH, MO, P, TAN)
*M*. *microphyllum* (MM)	West1	MMsta1	7	−23°28′14.3″S	43°45′54.0″E	Madagascar, Toliara province, south of Toliara, 5 km north of Grotte de Sarodrano (St. Augustin)	*M*. *Hanes* 496 (EMC, MICH, MO, P, TAN)
	West2	MMMad1	9	−23°04′03.3″S	43°36′58.0″E	Madagascar, Toliara province, north of Toliara off RN9 just south of Madiorano on a small dirt track. About 4 km to the east. White sands and calcareous rock	No voucher obtained, but see *N*.*M*. *Andrianjafy* 1900 (MO, P, TAN)
	West3	MMPK132	6	−23°20′17.7″S	43°53′01.5″E	Madagascar, Toliara province, east of Toliara on RN7 at PK28. North side of road	*M*. *Hanes 507* (EMC, MICH, MO, P, TAN)
Total		5	40				
	South1	MN2	9	−25°35′08.1″S	45°09′47.3″E	Madagascar, Cap Ste. Marie Special Reserve. On short road to grotte, about halfway. Grove of trees on light red soil	*M*. *Hanes* 484 (EMC, MICH, MO, P, TAN)
	South2	MNfc	7	−25°34′17.4″S	45°23′E	Madagascar, Road to Faux Cap from Cap Ste. Marie Special Reserve on white sand, on dunes close to the sea	M. Koopman 396 (MO, P, TAN, WIS)
*M*. *nodulosum* (MN)	East1	MNman20	10	−25°03′39.5″S	46°38′E	Madagascar, Fort Dauphin area, 20 km north of Ambosary en route to Tranomaro east side of road	*M*. *Koopman* 407 (P, TAN)
	East2	MNman40	4	−24°41′54.2″S	46°26′35.2″E	Madagascar, Fort Dauphin area, 42 km north of Ambosary en route to Tranomaro east side of road	*M*. *Koopman* 265 (MO, P, TAN, WIS)
	West	PK13MN	7	−23°24′49″S	43°46′48″E	Madagascar, Toliara province, south of La Table close to km marker 17, from Toliara, along roadside in dry scrub forest	*M*. *Koopman* 279 (MO, P, TAN, WIS)
Total		5	37				
Total *Megistostegium*	3	12	89				
*Humbertiella*							
*H. decaryi*			1	−23°04′03.3″S	43°36′58.0″	Madagascar, Toliara province, north of Toliara off RN9 just south of Madiorano on a small dirt track. About 4 km to the east. White sands and calcareous rock	*M*. *Koopman* 505 (MO, P, TAN, WIS)
*H. henrici*			1	−25.33′04″S	45.09′26″	Madagascar, Cap Ste. Marie Special Reserve. 6 km from Cap (about 3 km from ANGAP office) on road into protected area, dry scrubland	*M*. *Koopman* 250 (MO, P, TAN, WIS)
Total *Humbertiella*			2				

Library preparation and sequencing of RAD markers from genomic DNA was performed by Floragenex Inc. (Portland, Oregon, USA) using the single restriction enzyme *Pst1* and sample‐specific barcodes. Individuals were run multiplexed on two lanes of Illumina Hi‐Seq 4000 platform at the University of Oregon (Eugene, Oregon, USA) for 75 cycles to generate 100bp, single‐end reads. Outgroups include two species from the closely related, endemic genus *Humbertiella* Hochr. (*H*. *decaryi* and *H*. *henrici*).

### 2.2 RADseq bioinformatics

Raw RADseq data was analyzed with the Python analytical pipeline ipyrad v.0.9.41 (Eaton, 2014; Eaton & Overcast, 2020). Demultiplexed and filtered reads were used to generate homologous de novo RAD loci. Reads were clustered within samples at 85% sequence similarity, and a minimum read depth of 6, into de novo loci. Loci were then clustered across samples at 85% sequence similarity and processed into five assemblies. We produced two broad categories of sequence assemblies for analyses: (1) one “phylogenomic” assembly was constructed for phylogenetic analyses among the three species, where all loci were shared among at least 12 ingroup and outgroup taxa (89 *Megistostegium* samples, two outgroup samples) and (2) four “population genomics” assemblies were constructed for phylogeographic analyses within the two species with wide distributions. Two assemblies were constructed where all loci were shared among at least 30% of ingroup individuals (MM: 40 *Megistostegium microphyllum* samples and MN: 33 *M*. *nodulosum* samples). Two additional “population genomics” assemblies were constructed where all loci were shared among at least 30% of ingroup taxa and outgroup taxa (assemblies MMOG and MNOG). We present datasets with relatively low sample coverage thresholds (and higher levels of missing data) as they allowed for the inclusion of more loci. Several recent RADseq studies suggest that such sampling schemes are particularly useful in resolving relationships between recently diverged populations (Eaton et al., 2017; Park & Donoghue, 2019; Rubin et al., 2012; Tripp et al., 2017). Demultiplexed sequence data and SNP variants for phylogeographic analyses are available on the NCBI Short Read Archive and Dryad Digital Repository, respectively.

### 2.3 Phylogenetic tree estimation

Maximum‐likelihood analyses were conducted on the three assemblies with outgroups (phylogenomics; population genomics: MMOG and MNOG) using RAxML version 8.2.1 (Stamatakis, 2014) and implemented on the CIPRES Science Gateway Portal (https://www.phylo.org/portal2/home.action) under a GTR+CAT substitution model and with 100 rapid bootstrap replications to assess support.

### 2.4 Population genomic analyses

We examined population structure in *M*. *microphyllum* and *M*. *nodulosum* using the 30% “population genomics” assemblies (MM and MN) containing only ingroup taxa and one randomly selected SNP per RAD locus, implemented in structure v.2.3.4 (Pritchard et al., 2000) in the analysis kit of ipyrad. We executed 10 structure runs per *k* value (1–10), with each run having a burn‐in of 100,000 followed by 1,000,000 MCMC replicates. Results across runs were used to calculate the most probable number of clusters (*K*) using the method of Evanno et al. (2005).

### 2.5 Ecological Niche models

#### 2.5.1 Bioclimate

We used ecological niche models to understand the ecological distributions of each species and groups of populations (regions) to describe the extent of niche differentiation across the distribution of *Megistostegium*. We assembled point data by pooling georeferenced databases of distribution records from the herbaria of the Missouri Botanical Garden (MO) and the Muséum National d’Histoire Naturelle (P) and field collections of M. Hanes. Localities from herbaria were only included when coordinates were accurate, or locality descriptions were sufficiently detailed to infer coordinates. A total of 131 georeferenced localities (Figure 2c; *M*. *microphyllum*‐ 62 specimen records; *M*. *nodulosum*‐ 56 specimen records; *M*. *perrieri*‐13 specimen records) were used to construct ecological niche models. The ranges of each species and adjusted ranges with ecological niche modeling were initially produced using climate data for each of the 19 BIOCLIM variables downloaded from the WorldClim (version 1.4) variables at a 30‐sec (ca. 1 km^2^) spatial resolution (Hijmans et al., 2005). Datasets were clipped to the regional extent of Madagascar and projected to the GCS WGS 1984/UTM Zone 38S coordinate system using ESRI arcgis (v9.2). Pearson’s correlation tests were performed in the ARCMAP plugin SDMtoolbox v2.4 (Brown et al., 2014, 2017) to remove highly correlated bioclimate variables (Pearson *r* ≥ .70). Ecological niche models were calculated using both MaxEnt (Phillips et al., 2004, 2006) and the BioClim algorithm in the R package enmtools (Warren et al., 2019, 2021). Models were estimated from the average of 10 replicates and model performance was estimated using 25% of the points to test the model; the remaining points were used for training. We used the Area Under the Curve (AUC), calculated for testing data, to further assess model performance (Phillips et al., 2006). Once we verified that models were able to predict occurrence data better than random for species models with contemporary data (observed average by AUC =0.988 (MaxEnt); 0.87 (BioClim)), we predicted the geographic distribution of *Megistostegium* species in the past and into the future. We produced models for each species and projected them onto historical climate layers from the past (Last Glacial Maximum; ~22,000 ya), and the future (yr 2070; rcp45; CCSM4 Model; Gent et al., 2011); all variables were downloaded from WorldClim (past and future temporal conditions used downscaled and calibrated CMIP5 data with a 2.5 min and 30 sec spatial resolution, respectively; Hijmans et al., 2005). We projected our models into the future to gain insight into how predictions of drier and hotter conditions in the region (Hannah et al., 2008) will affect the distribution and conservation management of the more widely distributed species of *Megistostegium*, *M*. *microphyllum*, and *M*. *nodulosum*.

Because ecological niche models might conceal important spatial variation within the distribution of individual widespread species, we built ENMs using BioClim at the regional level to examine niche divergence at a finer scale. These ENMs were constructed in enmtools and model performance was evaluated with AUC. We used major lineages inferred by the phylogeographic analyses to partition populations into regions. Though the precise geographic boundaries of each lineage are unknown, some localities between regions were removed to perform niche modeling analyses. A total of 94 georeferenced localities (*M*. *microphyllum*‐ 45 specimen records, 10–19 records/region; *M*. *nodulosum*‐ 49 specimen records, 10–25 records/region) were used to construct regional distribution models.

#### 2.5.2 Soil

Ecological tolerances of each species with respect to soil were separately explored by producing ecological niche models, calculated in maxent v3.3.3e (Phillips et al., 2004, 2006) as the average of 10 replicates, using 18 soil groups from TAXOUSDA (downloaded from SoilGrids September 2017; ISRIC—World Soil Information; Hengl et al., 2017). Model performance was estimated using 25% of the points to test the model and the remaining points were used for training. Model performance was assessed with Area Under the Curve (AUC). No soil data was used in subsequent hypothesis tests.

### 2.6 Niche overlap and equivalency

Niche overlap values were calculated with BioClim models in the R package enmtools (Warren et al., 2019, 2021) using contemporary, noncorrelated bioclimate data for two sets of comparisons, (1) between species and (2) between each region encompassed by a species (East, West, South). Niche overlap values were evaluated using two standard metrics, Schoener’s *D* (1968) and Hellinger’s *I* (Warren et al., 2008), which produce values between 0 (no overlap between niche models) and 1 (identical niche models). Two randomization tests were carried out in enmtools (Warren et al., 2019) to further evaluate niche conservatism between species and regions using contemporary, noncorrelated bioclimate data. Niche identity tests aim to determine whether a pair of species (or regional lineages) has equivalent niches (Warren et al., 2010). Essentially, ecological niche models generated with point localities are compared with pseudoreplicate models generated with randomly selected points from a pool of actual point localities. Paired niche comparisons are deemed not equivalent if the overlap values are significantly different than those of the null distribution (*p* ≤ .05). Symmetric background similarity tests, instead, compare differences in the environmental background of paired‐comparisons to discern whether two species are more or less similar than expected by chance. Replicates were constructed from the background points supplied for each species (or region), with a 20 km radius circular buffer drawn around each point; 1000 background points were returned for each model. For each paired‐comparison, the niche model for one lineage is compared to pseudoreplicate models generated by randomly sampling the geographic range of its paired lineage (Warren et al., 2010). We used an area with a prediction threshold ≥0.25 based a binomial test of omission for the generated models. Niches are considered more similar than expected based on their background environments when the observed value of *D* is larger than the upper 95% confidence limit of the null distribution (a pattern of niche conservatism). Likewise, a pattern of niche divergence is supported when the observed *D* is smaller than the lower 95% confidence limit of the null distribution. 100 pseudoreplicates were created for each paired‐comparison for each test. *D* and *I* values were calculated for each pseudoreplicate model and the distribution of these values was compared to the niche overlap values calculated for the actual data.

## 3 RESULTS

### 3.1 RADseq bioinformatics

In total, more than 6.39 × 10^8^ single‐end reads were generated by Illumina sequencing of the RADseq library. After filtering low quality reads, defined as a read with more than 5 low quality base calls and a default phred Qscore offset of 33, more than 99% of reads were retained. On average, 7.02 × 10^6^ reads were sequenced per sample (range = 3.52 × 10^5^–1.87 × 10^7^ reads). The “phylogenomic” assembly recovered a total 24,481 RAD‐loci, 48,865 parsimony‐informative sites and 69.46% missing data (Table 2). The number of RAD‐loci recovered in the “phylogeographic” assemblies was 32,401, 23,863, 51,485, and 38,612 for MM, MMOG, MN, and MNOG, respectively (Table 2). These datasets contain between 40% and 48% missing data.

**TABLE 2:** Summary statistics for iPYRAD assemblies

	Ingroup	Outgroup	N reads	Min # ind. for which all loci were shared across taxa	Loci	PIS	Missing data	Average reads sequenced /sample	Minimum reads in a sample	Maximum reads in a sample
Phylogenomic assembly	89	2	6.39 × 10^8^	12	24,481	48,865	69.46%	7.02 × 10^6^	3.52 × 10^5^	1.87 × 10^7^
Population genomic assemblies										
*M*. *microphyllum* (MM)										
MMOG	40	2	3.05 × 10^8^	12	23,863	57,995	46.96%	7.27 × 10^6^	9.36 × 10^5^	1.44 × 10^7^
MM	40	0	2.86 × 10^8^	12	32,401	81,618	40.75%	7.16 × 10^6^	9.36 × 10^5^	1.44 × 10^7^
*M*. *nodulosum* (MN)										
MNOG	33	2	1.24 × 10^8^	10	38,612	82,036	48.46%	3.57 × 10^6^	1.6 × 10^5^	8.43 × 10^6^
MN	33	0	1.16 × 10^8^	10	51,486	109,638	44.37%	3.52 × 10^6^	1.6 × 10^5^	8.43 × 10^6^

### 3.2 Phylogenetic tree estimation

**FIGURE 3:**
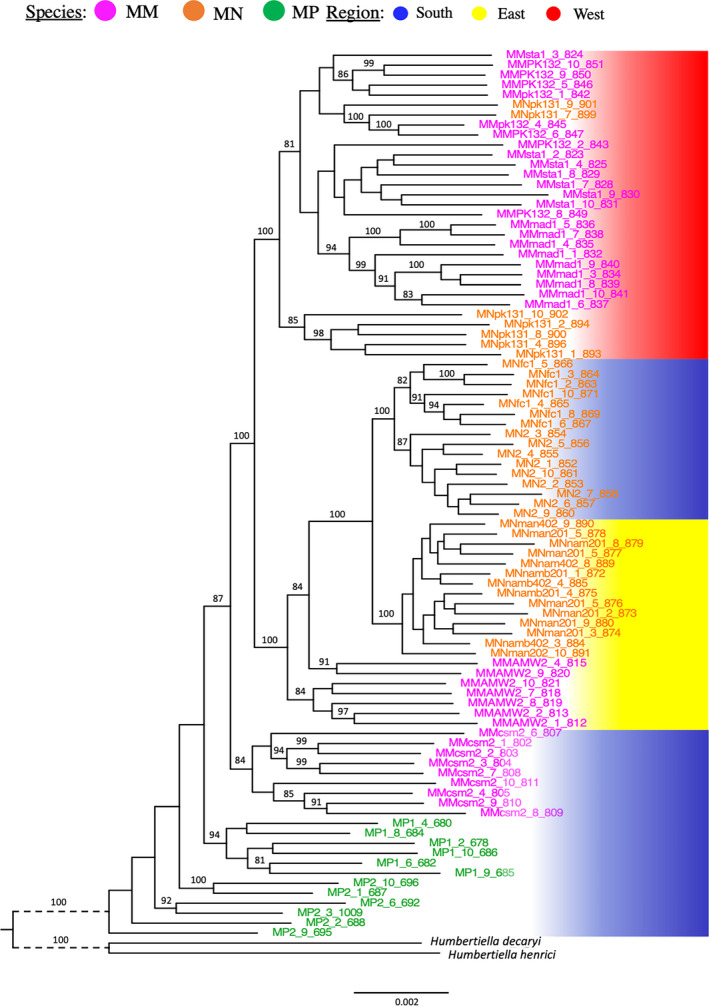
Maximum‐likelihood estimate of phylogenomic relationships in *Megistostegium* (‘phylogenomics’ assembly) inferred from concatenated RAD‐loci. Tips are colored by species, bars at right are colored by region. Only bootstrap values higher than 80 are shown

The ML approach of the “phylogenomic assembly” with outgroups recovered a well‐resolved tree, strongly structured by geography, that supports no species as monophyletic (Figure 3). Individuals of *M*. *microphyllum* and *M*. *perrieri* from the southern region together represent the earliest branching lineages and are distantly related to southern representatives of *M*. *nodulosum*. The western region (red) is the only monophyletic region and only two populations sampled from this region are reciprocally monophyletic. In the East (yellow), all *M*. *nodulosum* individuals form a monophyletic group but populations are not monophyletic. Eastern members of *M*. *microphyllum* form a grade with eastern and southern *M*. *nodulosum*.

### 3.3 Population genomic analyses

**FIGURE 4:**
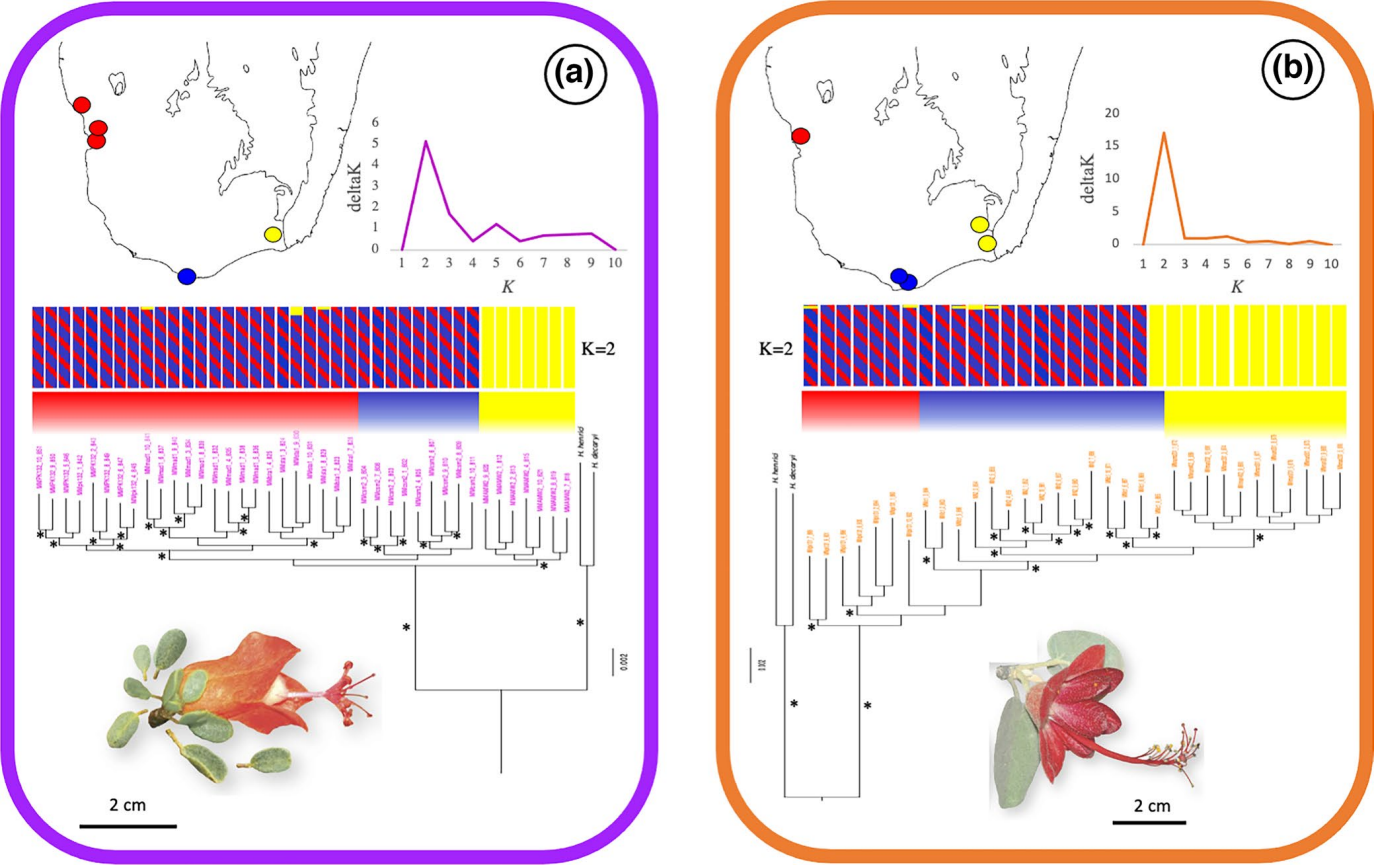
Maximum‐likelihood phylogeny, deltaK plots, structure plots, and population localities sampled for the two species groups found in more than one region. (a) *M*. *microphyllum*, and (b) *M*. *nodulosum*. Top—Population localities and deltaK plots summarized results across ten structure runs to calculate the most probable number of clusters (*K*) using the method of Evanno et al. (2005). Middle—structure plot for each species using a single SNP per locus using ‘Population genomics’ assemblies. Bottom—ML phylogenetic estimate of concatenated RAD‐loci using “Population genomics” with outgroup assemblies. * = bootstrap values higher than 80. Representative images provided for each species. (Photo credit: M. Hanes)

“Population genomics” assemblies for *M*. *microphyllum* and *M*. *nodulosum* produced fairly well‐resolved trees that are highly structured by geography (Figure 4). In the *M*. *microphyllum* tree, all individuals are strongly structured by region and all populations are exclusively monophyletic (Figure 4a). All populations in the West are sister to the southern population, which are in turn sister to the population in the East. The *M*. *nodulosum* tree reflects regional signal but with less support and no population monophyly (Figure 4b). All individuals from the East form a well‐supported clade that is sister to some individuals in the South. The remaining individuals from the South form a grade with individuals from the West. Simulations in structure assigned individuals in each species to two clusters that correspond to an East versus South/West cluster (Figure 4; Table 3).

**TABLE 3:** Summarized results across ten structure runs to calculate the most probable number of clusters (*K*) using the method of Evanno et al. (2005)

MM phylogeographic assembly			MN phylogeographic assembly		
*K*	delta*K*	lnP(*K*)	*K*	delta*K*	lnP(*K*)
1	0.000	0.00	1	0.000	0.00
2	5.173	124.73	2	17.053	83.94
3	1.540	27.11	3	0.905	−0.08
4	0.424	1.60	4	0.968	−4.83
5	1.246	11.61	5	1.171	−10.30
6	0.402	−5.81	6	0.377	−5.59
7	0.710	1.59	7	0.517	−10.56
8	0.734	−12.67	8	0.056	−5.78
9	0.781	0.27	9	0.534	−5.36
10	0.000	−11.93	10	0.000	−1.56

### 3.4 Ecological Niche models

#### 3.4.1 Bioclimate

**FIGURE 5:**
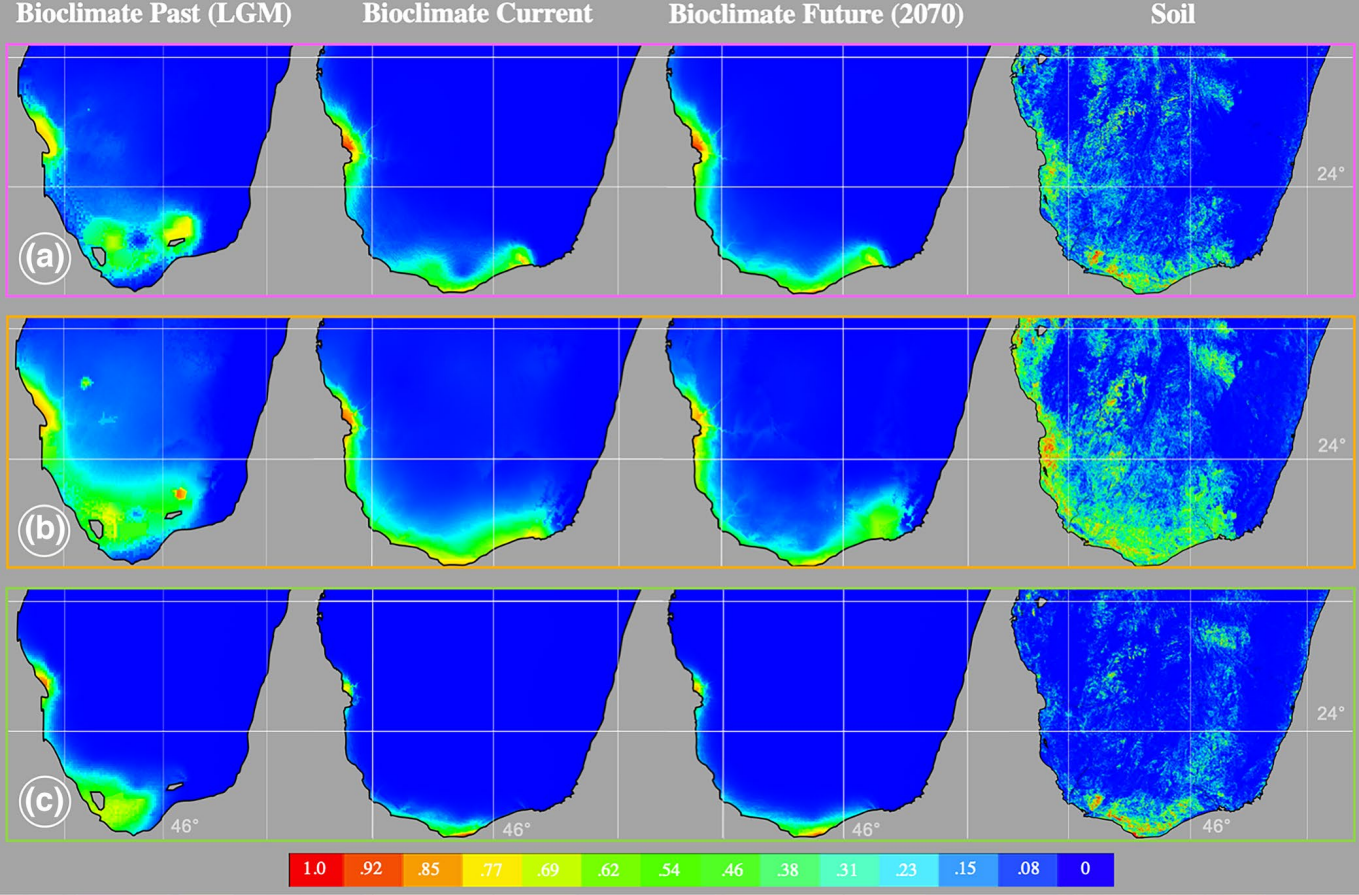
Ecological niche models based on five noncorrelated bioclimate layers of past, present, and future distributions and, separately, ENMs based on 18 soil layers, for each species using MaxEnt. (a) *M*. *microphyllum*, (b) *M*. *nodulosum*, and (c) *M*. *perrieri*. LGM (Last glacial maximum)

After highly correlated bioclimate variables were removed, five WorldClim variables were included (annual mean temperature (BIO1), mean diurnal range (BIO2), isothermality (BIO3), annual precipitation (BIO12), and precipitation seasonality (BIO15)). Species ENMs generated across the three time periods had average AUC values of 0.99 (with MaxEnt) and 0.87 (with BioClim), suggesting robust models. Comparisons between past and current projections suggest a contraction in the distribution of all species since the last glacial maximum (Figure 5a–c). Models ~50 years into the future predict little change in the distribution of all species. Regional ENMs had average AUC values of 0.785 (with MaxEnt) and 0.839 (with BioClim).

#### 3.4.2 Soil

ENMs generated in MaxEnt with soil layers had AUC values ≥0.953. All species have Arenosols and Ferralsols heavily represented in their soil layers, corresponding to unconsolidated quartz sand grains in the form of young and old dunes, as well as red sand outcrops. Soil distribution models, however, also suggest that the three species differ rather significantly in the soils they can tolerate (Figure 5a–c). *M*. *microphyllum* and *M*. *nodulosum* share Lixisols (ordinarily recovered in the driest soils in humid climates) and Luvisols (characteristic of soils in flat landscapes). *M*. *perrieri’s* limited distribution as predicted by bioclimate is further supported by strict soil composition (Figure 5c), for example, *M*. *perrieri* is the only species with Leptosols highly represented. Leptosols have high gravel content and a shallower profile when compared with other soil layers.

### 3.5 Niche overlap and equivalency

#### 3.5.1 Species comparisons

**FIGURE 6:**
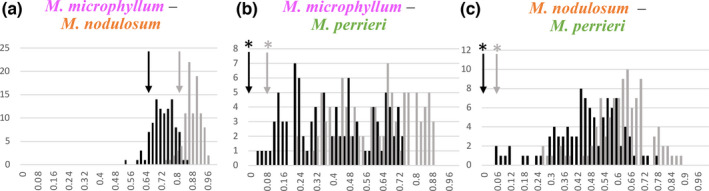
Histograms showing results of the background tests performed with 100 replicates for each species pair in *Megistostegium* using contemporary, noncorrelated bioclimate data. (a) *M*. *microphyllum*–*M*. *nodulosum*, (b) *M*. *microphyllum*–*M*. *perrieri*, and (c) *M*. *nodulosum*–*M*. *perrieri*. Black and gray bars represent background null distributions of *D* and *I* values, respectively. Arrows represent observed values of *D* and *I* (*p < .02)

All species pairs have nonequivalent ENMs (Table 4). Niche similarity assessed by the niche overlap metric was greater between *M*. *microphyllum* and *M*. *nodulosum* (*D* = 0.654/*I* = 0.836) than between the other species comparisons (*M*. *microphyllum*–*M*. *perrieri* (*D* = 0.01 /*I* = 0.09); *M*. *nodulosum*–*M*. *perrieri* (*D* = 0.006/*I* = 0.07; Table 4)). Niche identity and symmetric background tests indicate that the null hypothesis cannot be rejected for *M*. *microphyllum*–*M*. *nodulosum* (Figure 6a). The null hypothesis was rejected for *M*. *microphyllum*–*M*. *perrieri* and *M*. *nodulosum*–*M*. *perrieri*, as all observed values of *D* and *I* were significantly smaller that the null distributions (Figure 6b,c).

**TABLE 4:** Results of niche overlap analyses for pairwise species comparisons

Species	Niche Overlap		Identity test		Background test		Inference
	*D*	*I*	*D*	*I*	*D*	*I*	
MM—MN	0.653	0.836	*p *= .168	*p* = .316	*p* = .65	*p* = .93	Null
MM—MP	0.01003275	0.09121992	*p *< .01	*p* < .01	*p* < .02	*p* < .02	Divergent
MN—MP	0.00635937	0.07413763	*p *< .01	*p* < .01	*p* < .02	*p* < .02	Divergent

Note

Identity and background tests are based on 100 replicates. *D* = Schoener’s *D*; *I* = Hellinger’s *I*.

#### 3.5.2 Regional comparisons

Within a region all niche overlap values between species have nonzero values (Table 5). In the southern and eastern regions, all species pairs have small niche overlap values. In the West, *M*. *microphyllum* and *M*. *nodulosum* have higher values of niche overlap (Table 5). Background tests confirm that niches occupied between regions within a species are very different across southwestern Madagascar. The null hypothesis can be rejected for four out of five intraregional comparisons between species via background similarity tests (Table 5).

**TABLE 5:** Results of niche overlap analyses for pairwise regional comparisons

		Regions	Niche overlap		Background test	
			*D*	*I*	*D*	*I*
Intraregional	South	MMS—MNS	0.012	0.051	*p* = .237	*p* = .039
		MMS—MPS	0.064	0.241	*p* < .02	*p* < .02
		MNS—MPS	0.058	0.075	*p* < .02	*p* < .02
	West	MMW—MNW	0.575	0.697	*p* < .02	*p* < .02
	East	MME—MNE	0.010	0.043	*p* < .02	*p* < .02
Interregional	Intraspecific	MMS—MME	0.000	0.000	*p* < .02	*p* < .02
		MMS—MMW	0.000	0.000	*p* < .02	*p* < .02
		MME—MMW	0.000	0.000	*p* < .02	*p* < .02
		MNS—MNE	0.000	0.000	*p* < .02	*p* < .02
		MNS—MNW	0.000	0.000	*p* < .02	*p* < .02
		MNE—MNW	0.000	0.000	*p* < .02	*p* < .02
	Interspecific	MMS—MNE	0.000	0.000		
		MME—MNS	0.000	0.000		
		MME—MPS	0.000	0.000		
		MNE—MPS	0.000	0.000		
		MMS—MNW	0.000	0.000		
		MMW—MNS	0.000	0.000		
		MNW—MPS	0.000	0.000		
		MMW—MPS	0.000	0.000		
		MME—MNW	0.000	0.000		
		MMW—MNE	0.000	0.000		

Note

Background tests are based on 100 replicates. *D* = Schoener’s *D*; *I* = Hellinger’s *I*.

Remarkably, within a species metrics of niche overlap indicate no overlap between regions across a species’ range (i.e., niche overlap is zero for every intraspecies interregional comparison) (Table 5). The null hypothesis is rejected for all comparisons between species via background similarity tests (Table 5). A similar pattern is observed for every interspecies, interregional comparison.

## 4 DISCUSSION

The phylogenomic tree comprising all individuals representing the three species of *Megistostegium* indicates that *M*. *perrieri* is the earliest diverging species. This prostrate and shrubby species is known only from the most southern distribution of the genus, on windy cliffs of the Mahafaly Plateau at Cap Sainte Marie and Lavanono. *M*. *perrieri* has the smallest distribution of any species and grows on unique, calcareous outcrops. Bioclimate niche models from the past suggest a more widespread distribution for this species; however, when we include patterns suggested by our soil models, we conclude that *M*. *perrieri* has likely been restricted in its distribution since its origin. The next diverging clade contains all individuals of *M*. *microphyllum* collected from the southern region (growing in close proximity with individuals of *M*. *perrieri* at Cap Sainte Marie (population MP1)).

Water availability is clearly important in *Megistostegium* and all dry adapted plants thriving in the spiny thickets. Annual rainfall gradients on the island decrease from north to south and from east to west (Koechlin, 1972) with coastal southwest Madagascar receiving between 400 and 300 mm per year (Donque, 1972; Serele et al., 2019; von Heland & Folke, 2014). The dry season consistently lasts between 6 and 9 months, though long lasting droughts of 12 months with no rain are not unusual (von Heland & Folke, 2014). It has been posited that plants in the spiny thickets may rely on coastal fog (Dewar & Richard, 2007). Groundwater might also be a very important source of water and estimates suggest moderate to high groundwater potential throughout the distribution of *Megistostegium*, with high potential to the eastern and western extremes of the genus (Serele et al., 2019). Such differences in water potential at the extremes of the distribution of *Megistostegium* might act to further isolate and differentiate populations between the three regions. Rivers throughout the region are mostly ephemeral, at least in contemporary times, and flow only during the short rainy season. It is thus interesting that the geographically structured patterns uncovered in all RADseq analyses can be partially explained by the presence of rivers. The distribution of *Megistostegium* is bisected by four rivers: three rivers between the west and the southern regions and one river between the southern and the eastern regions.

The closely related *M*. *microphyllum*–*M*. *nodulosum* species pair has yet to obtain exclusive species monophyly, though we recover regional and population monophyly in most cases within and between species. With such recent divergence events, one would expect such a species pair to have more niche overlap than either species might have with an older lineage (Anacker & Strauss, 2014), and indeed we observe substantially more niche overlap between *M*. *microphyllum* and *M*. *nodulosum* than any comparison with *M*. *perrieri*. In addition, permutation tests reveal a pattern consistent with niche divergence between *M*. *perrieri* and the other two species. A second expectation from recently diverged taxa with largely overlapping distributions, like *M*. *microphyllum* and *M*. *nodulosum*, is that ecological niche differentiation might not yet be discernable (Peterson, 2011). While such a conclusion appears possible at the species level across their entire ranges (the null hypothesis was not rejected for this species pair), tests at the regional level based on ecological niche modeling instead suggest that in the East and West *M*. *microphyllum* and *M*. *nodulosum* have highly significant divergent niches. Furthermore, *M*. *microphyllum* appears to be restricted to unconsolidated sands and dunes on the coast while *M*. *nodulosum* can tolerate a variety of soils, including tertiary limestone, further inland that are inhospitable to *M*. *microphyllum*.

When we compare models built with only southern occurrence points, we recover a similar pattern: niche overlap exists among all three species, but it is remarkably small, and again significantly smaller than expected in comparisons between *M*. *perrieri* and the other species. *M*. *microphyllum* and *M*. *nodulosum* share a very small niche overlap value (though the null hypothesis was not rejected) at this site when compared across the distribution of the genus. Data presented here suggest that each species in the South has discrete ecological tolerances across a small distance. Individuals from the three species collected from Cap Sainte Marie are generally reciprocally monophyletic and well supported, further suggesting that these taxa are discrete genetic entities in the South, despite evidence for incomplete reproductive isolation (Koopman, 2011; Koopman & Baum, 2010). Stratigraphy and geochronology have been well characterized at a nearby site (Faux Cap) in the South (Battistini, 1964; Mahé & Sourdat, 1972) and are broadly divisible into three major stratigraphic units: red sand outcrops more inland, older sands dunes and younger sands dunes. The thickness of the formations decreases from south to north (Tovondrafale et al., 2014). A small study conducted at Cap Saint Marie suggests that the three species of *Megistostegium* grow in different soil depths (Koopman, 2008).

The terrain across the distribution of *Megistostegium* is generally flat, differing between 0 and 2.5 degrees (in altitude) across the landscape (Serele et al., 2019), though an important exception is a series of plateau areas throughout the spiny thickets that are separated by the main river basins, including the cliffs at Cap Saint Marie. The combined bioclimate, topographical, sedimentary, and soil complexity potentially provide many avenues for diversification that could drive and maintain the distinct genetic lineages we uncover that are concurrent with morphological species at this site.

In the East, *M*. *microphyllum* and *M*. *nodulosum* are genetically distinct: in the population genomics trees eastern clades are monophyletic and well supported and further supported in structure results. Koopman and Baum (2010) similarly identified significant clustering of eastern individuals in *Megistostegium* relative to other sampled regions. Supporting strong genetic differentiation, *M*. *microphyllum* and *M*. *nodulosum* have negligent niche overlap in the East that is significantly smaller than expected. It is interesting that the eastern populations are so well differentiated, for while the Manambovo river divides the eastern region of *Megistostegium* from the rest of its distribution, this river (at least contemporarily) has no surface flow for most of the year (Aldegheri, 1972) and few other landscape features have been identified that might have driven the observed isolation between other regions. The eastern populations sampled in *Megistostegium* do, however, lie at the extreme eastern edge of the spiny thicket, in a small area that represents a unique and isolated desert with high local endemism (Aronson et al., 2018).

In the West, *M*. *microphyllum* and *M*. *nodulosum* are genetically distinct for the most part. An exclusively western clade is monophyletic on the phylogenomics tree and in the *M*. *microphyllum* population genomics tree. *M*. *microphyllum* and *M*. *nodulosum* have the largest niche overlap in this region than anywhere else in their range though the overlap is significantly smaller than expected by chance. Three rivers lie between the West and South regions and might add to the distinct population structure we observe in *M*. *microphyllum* in the western region. The Ohilahy River in the extreme northwestern distribution of *Megistostegium* was identified as a potential retreat corridor for mesic taxa during more arid times (Wilmé et al., 2006) and has also been hypothesized to act as a biogeographic barrier to two sister species pairs of plated lizards (Raselimanana et al., 2009) and to intraspecific population structure in one species of iguanid lizards (Chan et al., 2012). The influence of the remaining rivers that bisect the southern slopes and reside between the west and southern regions of *Megistostegium* has yet to be explored in other taxa.

### 4.1 Climate change and phylogeographic breaks

Ecological niche modeling with bioclimate data shows the southwestern coast has remained stable and hospitable to *M*. *microphyllum* over time and consistently highlights a break in the species’ distribution close to where the Linta and Menarandra rivers spill into the ocean. This break is also visible in soil models and is concurrent with genetic structure. Furthermore, we have identified significant changes in stratigraphy, carbon sequestration levels, and groundwater potential simultaneous with this discontinuity in the distribution of *M*. *microphyllum* (Besairie, 1967; Collignon, 1971; Grinand et al., 2009; Serele et al., 2019; Waeber et al., 2015). Such a landscape discontinuity is absent in the distribution of *M*. *nodulosum* in our bioclimate and soil ENMs and is not supported on the population tree nor in structure plots. We posit that such continuous ecological suitability for *M*. *nodulosum* along the west coast is at least partially driving the genetic interconnectedness between the southern and western regions in this species.

The Pleistocene and Holocene were dominated by aridification in southwestern Madagascar (Tovondrafale et al., 2014) concurrent with the expansion of xeric scrublands. Average sea level would have been substantially lower than contemporary levels (Lambeck et al., 2014) and presumably exposed significant new areas of coastline for these primarily coastal *Megistostegium* species. The drought tolerance of *Megistostegium* further allowed them to expand their range inland. During this proposed bidirectional range expansion, *M*. *microphyllum* and *M*. *nodulosum* may have become adapted to different ecological niches (and developed subsequent reproductive isolation). Post glaciation, as sea levels rose, we would expect local extinction and population displacement coincident with a new coastline. Likewise, inland communities would become more humid and dry adapted species would be forced west and southward. A notable contraction is visible in the distributions of *Megistostegium* species from the last glacial maximum to current conditions. Such a scenario provides evidence for the largely overlapping contemporary distributions we observe within the morphologically distinct species of *Megistostegium*. Paleoclimatic oscillations have presumably been important in the evolution of *Megistostegium* in other ways as well. We posit that expanded arid regions in the past promoted range expansion and higher connectivity throughout the region. Rivers running in more humid times likely acted to isolate populations of arid‐adapted *Megistostegium* driving the regional structure we observe today.

Since 1960, southwestern Madagascar has gotten warmer (by 0.2°C) and drier (40 mm less/avg month) (Tadross et al., 2008). In the future, temperatures are predicted to increase another 1.1 to 2.6°C and the region is expected to undergo further aridification (Hannah et al., 2008) with the greatest warming and drying expected in the southwest, along the coast. Under these scenarios, models consistently predict range expansion for plant species throughout the southwest (Hannah et al., 2008; Schatz et al., 2008). Distribution modeling into the future suggests little change in the distribution of *Megistostegium*. Our models suggest that *M*. *perrieri* may expand slightly in the future, but this is extremely unlikely given its specific, extreme ecological requirements. Such stable distribution models through time might seem to be good news for the future of the genus, however, we have noted population extinction in *Megistostegium* across the range of the genus over time (Hanes Pers. Obs) and only a small proportion of the distribution of *Megistostegium* is within the current protected area network in Madagascar. *Megistostegium* species are further used in several ethnobotanical practices and prized for its strong wood (Koopman, 2011). In 2011, we assigned the species of *Megistostegium* the following IUCN designations: Near threatened (*M*. *nodulosum*), Vulnerable (*M*. *microphyllum*),and Endangered (*M*. *perrieri*). For these reasons, it is clear that the future ecological niche models alone will not accurately predict the future of the genus. In addition, the dry, spiny thickets of southwestern Madagascar have the highest rate of deforestation on the island. Thirty percent of the forest was lost between 1970 and 2000 (Brinkmann et al., 2014; Harper et al., 2007) and studies estimate that between the years 2000 and 2005 these forests lost between 0.42 and 1.1% of forest/year (Brinkmann et al., 2014; MEFT et al., 2009). Unfortunately, the remoteness of the dry, spiny thickets combined with its harsh climate continues to elude conservation priority in these extraordinary plant communities (Moat & Smith, 2007).

## 5 CONCLUSIONS

Phylogeographic data from xeric habitats in southern Madagascar remain scarce and few hypotheses exist as to how such extraordinary species diversity in the spiny thickets was created. This work represents the first study to combine ecological niche models with phylogeographic analyses to understand plant diversification in the Malagasy southwest. We find evidence for the roles of bioclimate, soils, and recent climate change in shaping phylogeographic structure in *Megistostegium*. Our work further highlights the heterogeneity of precipitation and temperature throughout this semiarid region and we identify strong genetic structure across the distribution of *Megistostegium* coincident with bioclimate and low elevation rivers. Distribution modeling into the past supports the influence of paleoclimate oscillations that allowed two species of *Megistostegium* to expand their distributions in the arid Pleistocene and Holocene and become isolated across inhospitable mesic areas. Such range shifts could be a major driver of differentiation across southern Madagascar and should be explored further in other taxa. We also identified a new and potentially important biogeographic break in southwestern Madagascar. In the future it will be interesting to look for genetic structure across the rivers that bisect southwestern Madagascar, the distinct bioclimatic regions we uncovered, and also the biogeographic break that we identified in a diversity of taxa across this region. We also hope to employ a variety of population genomic analyses in the future to incorporate gene flow and historical demography to better understand the evolution of these species.

## CONFLICT OF INTEREST

The authors have no conflict of interest.

## AUTHOR CONTRIBUTIONS

**Margaret M. Hanes:** Conceptualization (lead); Data curation (lead); Formal analysis (lead); Funding acquisition (lead); Investigation (lead); Visualization (lead); Writing – original draft (lead). **Susan Shell:** Formal analysis (equal); Writing – review & editing (equal). **Tahsina Shimu:** Investigation (equal); Writing – review & editing (equal). **Clarissa Crist:** Formal analysis (equal); Writing – review & editing (equal). **Salima Machkour M’Rabet:** Formal analysis (equal); Writing – review & editing (equal).

## Data Availability

The demultiplexed sequence data that support the findings of this study are openly available on the NCBI Short Read Archive: PRJNA731041. Additional openly available data are available in the Dryad Digital Repository at https://doi.org/10.5061/dryad.w9ghx3fqn.
